# A Polymer-Biologic Hybrid Hernia Construct: Review of Data and Early Experiences

**DOI:** 10.3390/polym13121928

**Published:** 2021-06-10

**Authors:** Michael Sawyer, Stephen Ferzoco, George DeNoto

**Affiliations:** 1 Department of Surgery, Oklahoma State University, Comanche County Memorial Hospital, Lawton, OK 73505, USA; 2 Department of Surgery, Atrius Health, Dedham, MA 02026, USA; steven_ferzoco@atriushealth.org; 3 General Surgery Department, St. Francis Hospital, Roslyn, NY 11576, USA; George.Denoto@chsli.org

**Keywords:** reinforced tissue matrix, decellularized extracellular matrix, synthetic mesh, ventral hernia repair, inguinal hernia repair, hiatal hernia repair

## Abstract

Surgical mesh reinforcement of the human abdominal wall has been found to reduce the chance of recurrence in hernia repairs. While traditionally polymer meshes have been used in hernia repair, alternative mesh options have been engineered to prevent the inflammatory foreign body response invoked by polymers. A reinforced tissue matrix (RTM) mesh has been developed by embedding a polymer within a decellularized extracellular matrix. This combination has been attributed to the recruitment of host cells, a pro-healing response, and attenuation of the foreign body response. This has been observed to lead to the regeneration of functional tissue within the repair site that is reinforced by the polymer to offload abdominal pressures over time. This manuscript presents the review of OviTex, an RTM, in several types of hernia repair. The authors have found that the use of RTM in hernia repair is effective in preventing foreign body response, promoting wound healing, and providing reinforcement to lower the risk of hernia recurrence.

## 1. Introduction

### 1.1. Abdominal Wall Hernia and Treatment Options

Abdominal wall hernias are common clinical entities and represent a major source of disability and morbidity in patients. Until the mid-20th century, primary suture repair, where the defect in the abdomen is closed with polymeric sutures, was the only available option for their surgical treatment. Reported recurrence rates with this approach were relatively high. The advent and clinical application of a synthetic polymer-based mesh represented a significant advance in the surgical treatment of hernias. The mesh is sutured into the defect area, placed within planes of muscle and fascia in the abdominal wall, acting as a scaffold to provide mechanical support and a structure for tissue to “scar” into to reduce the chance of recurrence. A study comparing suture to synthetic mesh-reinforced repair showed a reduction from 63 to 32% for synthetic mesh-reinforced repairs, and, for this reason, almost 90% of hernia procedures today involve some sort of mesh [1,2]. While this reduced recurrence, the repaired “scar” does not have the same dynamic qualities as human fascia [3]. It also became recognized that the application of synthetic mesh was also associated with its own set of serious complications, including chronic wound infections, adhesions to and erosion into intra-abdominal viscera, enterocutaneous fistulae, and others. Most of these complications can be attributed to the prolonged foreign body exposure inherent to the amount, density, and type of permanent synthetic polymer in the mesh.

### 1.2. Wound Healing

The implantation of any foreign material in a human body elicits a foreign body response, although the extent and severity are dependent on the amount of foreign material and, in the case of a mesh, the density of the structure [4,5,6,7]. The response is characterized by an inflammatory reaction followed by the recruitment of fibroblasts and myofibroblasts, whereby the first mainly lay down new collagen and the latter generate contractile forces [8,9]. In a typical wound healing sequence, the fibroblasts and myofibroblasts in the end undergo apoptosis, and the newly laid collagen remains. However, in the presence of a continued stimulus caused by a foreign body, such as a permanent synthetic mesh, they may remain active. In the event of prolonged foreign body exposure, host cells wall off the material with fibrotic tissue characterized by disorganized collagen and inflammatory cells [10,11,12]. The ongoing struggle has been to design a mesh that balances defect area support while reducing the inflammatory response.

### 1.3. Mesh Landscape

Over the years, the mesh landscape has evolved to address this issue with the creation of resorbable polymer and biologic mesh materials. Resorbable polymer materials have been developed in combinations of polyglycolic acid, polylactic acid, trimethylene carbonate, poly-4-hydroxybutyrate (P4HB), and other natural and synthetic polymers. Biologic materials are derived from mammalian (e.g., human, ovine, bovine, or porcine) source tissues taken from organs, such as the dermis, intestines, stomach, or bladder, and decellularized to retain the extracellular matrix as a scaffold for remodeling [13].

Polymeric meshes continue to be chosen for most hernia repairs due to their low cost and ability to withstand intrabdominal wall pressures, ensuring the defect does not re-herniate. These meshes are knit from polymer fibers forming porous structures classified based on their surface area density and pore size. Lightweight meshes are classified as less than 35 g/m^2^, midweight between 50 and 80 g/m^2^, and heavy weight greater than 80 g/m^2^ [14], with lightweight meshes yielding the lowest dose-related inflammatory response [4,15]. A balance of pore size is also important to create holes large enough to promote cell infiltration, limit adhesions, and reduce the dose of polymer, while also remaining small enough to provide strength and prevent bulging [15,16].

The most common construct is a knit mesh made of monofilament fibers and, less often, multifilament fibers. Multifilament fibers are used less due to the spaces between the fibers being large enough for bacteria, yet too small to allow white blood cells, preventing an immune response to fight the formation of a biofilm and slime, which can lead to surgical site infections (SSI) [17,18,19]. Incisional ventral hernia repair has a four times larger incidence of infection than inguinal/femoral or umbilical hernias [20]. For this reason, when multifilament fibers are used, they are typically constructed of a polymer with a relatively short resorption time, such as polyglycolic acid.

Synthetic meshes have also been combined with barriers to prevent some of the most common complications associated with the prolonged foreign body response, such as viscera adhesions. Seprafilm (Genzyme, Cambridge, MA, USA), made of hyaluronic acid and carboxymethylcellulose, is an anti-adhesive bioresorbable barrier coating first used for abdominal and pelvic laparotomy and later as a coating for Phasix and Ventralight. While the barrier reduced the chance of adhesions [21], it also prevented tissue ingrowth, limiting the ability of the mesh to create a fascia-like layer of tissue in the defect [22].

P4HB (Phasix) is one of the more promising fully resorbable meshes, undergoing hydrolysis and degrading within 12–18 months. Roth et al. recently published on its clinical performance from a prospective, multi-center single arm study showing a three-year recurrence rate of 18% [23]. While this recurrence rate is significantly less than early permanent repairs, it has demonstrated localization of histiocytic cells walling off the polymer fibers of the mesh resulting in comparable inflammatory levels to two other permanent synthetics at the late chronic time point in a full thickness abdominal wall repair model in non-human primates [24].

Biologic materials exist as an alternative to synthetic materials due to their inherently lower inflammatory response and resilience, particularly in the repair of infected or contaminated fields and other high-risk patients undergoing hernia repair [25]. These materials are harvested from xenogeneic or allogeneic organs to isolate the extracellular matrix (ECM), a three-dimensional structure possessing proteins and signaling molecules [13,26]. The biologic materials are processed to remove cells and DNA of the source species to create an immunologically inert scaffold for remodeling, though the quality and extent of this is dependent upon the processing methods [27,28]. While these materials show promise, unfortunately, they are expensive and due to the retention of the protein elastin, may stretch over time [29,30,31].

### 1.4. Ideal Mesh

The ideal mesh would possess the strength and handling of a polymer mesh and possess the proteins and signaling molecules of a biologic ECM to attenuate the foreign body reaction and promote wound healing. The creation of a polymer mesh loaded with proteins and signaling molecules from an ECM is a challenge not only in creation but also in cost, scalability, and clinical utility. Studies have been conducted to include these proteins within polymeric materials but have largely been limited to lab settings with only in vitro results. Furthermore, the clinical utility of these have not been proven beyond in vitro studies.

### 1.5. Hybrid Meshes

A potential alternative solution to this is the embedment of a polymer mesh in a biologic material forming a hybrid composite mesh. In a study by Liang et al., researchers showed that a polypropylene mesh can behave like a biologic material by embedding it in a biologic extracellular matrix, reducing the inflammatory response. The result showed a mature elastin content, sulfated glycosaminoglycan content, and collagen subtype III/I ratio. These findings were attributed to the recruitment of pro-healing M2 macrophages by the ECM rather than proinflammatory M1 macrophages [32,33,34,35]. Similar designs have also been made by embedding a polypropylene mesh within an acellular dermal matrix showing a reduction in adhesions, a common complication associated with synthetic meshes [29].

### 1.6. Reinforced Tissue Matrices

Two relatively new hybrid meshes, consisting of a polypropylene or polyglycolic acid mesh, interwoven within layers of ovine-derived ECM, have shown promise as a suitable alternative both preclinically and clinically. The hybrid device creates a three-dimensional scaffold, interweaving polymer throughout layers of ovine (sheep) forestomach (rumen)-derived ECM. The polymer provides strength and compliance comparable to that of the human abdominal wall [24,36,37,38], critical to offloading the wound during the acute healing phase. The layers of the ECM and the channels and pores both inherent to the structure of the ECM and created by the interwoven polymer promote fluid exchange and have been attributed to the rapid host cellular response and infiltration of the network [24]. This rapid recruitment is critical in the attenuation of the foreign body reaction to the polymer, surrounding the fibers with host cells, resulting in a functional tissue repair both directly around the polymer fibers and within the ECM, with no encapsulation of the fibers nor “scar”-like collagen as is seen in synthetic devices [24]. It is believed that these properties unique to reinforced tissue matrices provide an advantage over synthetics in the reduction of SSOs, infection, recurrence, and inflammatory response in comparison to purely polymeric materials.

**Figure 1:**
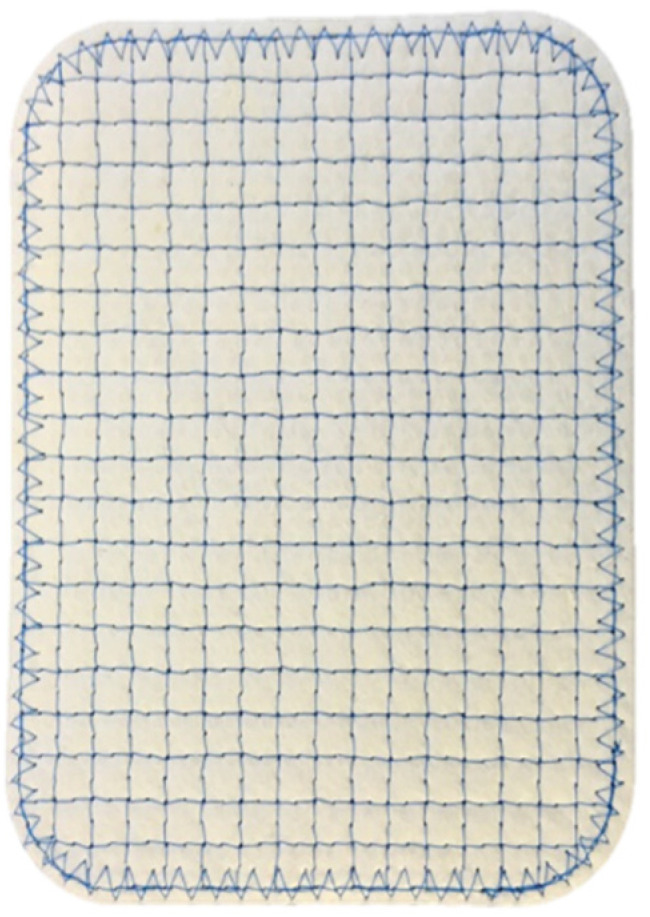
OviTex RTM mesh.

**Figure 2:**
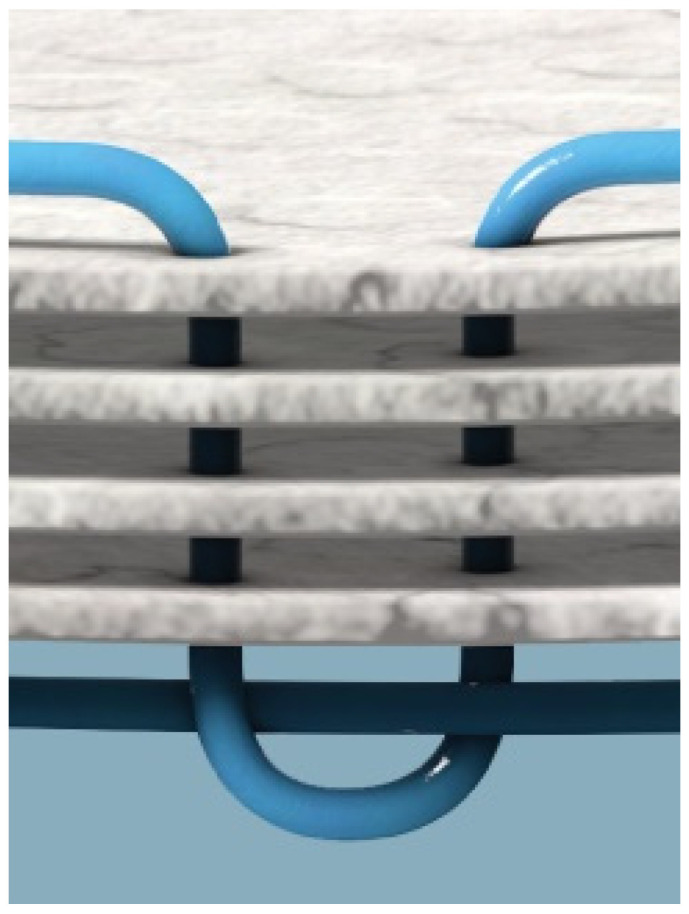
OviTex RTM cross section of four-layer device with polypropylene.

Reinforced tissue matrices (OviTex Core, 1S and 2S Resorbable and Permanent) are produced by interweaving polypropylene (PP) and polyglycolic acid (PGA) filament through layers of ovine (sheep) rumen, specifically the forestomach, as depicted in Figure 1 and Figure 2. The ovine forestomach is processed to isolate the propria submucosa from the tunica muscularis and lamina epithelia. The extracellular matrix (ECM) of the propria submucosa is gently decellularized via a proprietary processing method (Aroa Biosurgery, New Zealand) to give ovine forestomach matrix (OFM) [39]. OFM is composed of structural and functional proteins, including collagen I and III, fibronectin, glycosaminoglycans, elastin, fibroblast growth factor basic, laminin, and collagen IV [39]. A recent proteomic analysis has identified more than 150 different ECM proteins that exist in OFM [40], and the matrix structure is essentially identical to tissue ECM [41,42]. Reinforced tissue matrices are made in several configurations, primarily differing in the number of OFM layers (four to eight) as well as the amount and density of polymer. The four-layer device is engineered with either PP or PGA, interwoven as a 6 mm grid pattern, while the six- and eight-layer devices add an additional two layers to one or both sides with a less dense 25 mm PP or PGA interwoven grid to reduce the risk of adhesions. All devices are terminally sterilized with ethylene oxide prior to use. OviTex devices are cleared for hernia repair and soft tissue reinforcement and have been used since July 2016.

Review data for published works on RTM in ventral, hiatal, and inguinal hernias are presented to evaluate the effectiveness of RTMs in preventing foreign body response, promoting wound healing, and providing reinforcement to lower the risk of hernia recurrence.

## 2. Review of Reinforced Tissue Matrices in the Repair of Various Hernia Types

### 2.1. Ventral Hernia Repair

Ventral hernias are defects of the fascia abdominal wall, which allow for projection of abdominal organs and tissues through this wall [43]. Ventral hernia repair (VHR) commonly requires surgical intervention to place these protruding tissues and organs back into the abdominal cavity and close the defect in the abdominal wall [44]. While in some cases ventral hernia defects can be closed through suture alone, use of mesh supports has been shown to decrease incidence of recurrence [44]. As described above, several types of mesh are available in order to repair ventral hernias. The type of mesh used for repair is selected based upon durability, sterility, cost, foreign body reaction, host tissue ingrowth, patient comorbidity, surgeon preference, etc. [45]. The reinforced tissue matrix, OviTex, has shown promise in preventing recurrence in ventral hernia repair in preclinical and early clinical studies [24]. Recent studies have corroborated these early indications.

A single institution retrospective review by Parker et al. published in September of 2020 showed OviTex to be effective in high-risk patients who underwent VHR (Table 1) [46]. This study compared the surgical outcomes when OviTex was used to repair ventral hernias in high-risk patients compared to lower risk patients in which synthetic polypropylene mesh with a barrier was utilized [46]. Patient risk was determined based on hernia severity, classified on two different scales [46]. The modified Ventral Hernia Working Group (VHWG) grading classification system uses three grade levels to indicate hernia severity, grade 1 being the lowest risk, least severe hernias and grade 3 being contaminated, high-risk hernias [47]. The Center for Disease Control (CDC) wound classification scale ranks hernia severity from class I–IV, with class I being clean, less severe hernias and class IV being dirty/infected, high-risk hernias [48]. In this study, OviTex was used to repair ventral hernias in 50 consecutive patients with mostly VHWG grade 3 (61%) and CDC wound class III (61%) hernias [46]. Synthetic mesh was used to repair ventral hernias in 45 patients with mostly VHWG grade 2 (91%) and CDC wound class I (91%) hernias [46]. After 12 months, the hernia recurrence rate was lower in the OviTex group despite having a more challenging population than the synthetic group, 6 vs. 12% for OviTex and synthetic groups, respectively [46]. This was also true when recurrence rates were evaluated in a subset of patients who had developed a complication or infection at the surgical site, known as a surgical site occurrence (SSO). The rate of recurrence in this OviTex group was lower at 17% compared to the synthetic mesh group at 55%, a result that is counter-intuitive when considering SSOs typically increase the risk of recurrence [46]. This study indicates that recurrence rates in challenging patients, including those with SSOs, are lowered when OviTex is used compared to synthetic mesh. [46].

### 2.2. Hiatal Hernia Repair

Hiatal hernias occur when a portion or all of the stomach protrudes through a defect in the esophageal hiatus of the diaphragm, the portion of the diaphragm in which the esophagus passes from the thoracic to the abdominal cavity [49]. As with ventral hernias, surgical intervention is often needed to correct this defect and use of mesh decreases recurrence rate. Case studies have demonstrated that permanent synthetic meshes, used to repair these defects, can erode into the esophagus [50,51,52]. Reinforced tissue matrices could play a role in the repair of these hernias for surgeons.

Previously, Sawyer conducted a retrospective study to document their surgical experience in using resorbable OviTex to repair symptomatic hiatal hernias (Table 1) [53]. Surgical repair was performed on 25 patients who had a high incidence of comorbidities and 52% who had type III and IV hiatal hernias [53]. These hernias were considered high-risk as the anatomic classification scale for hiatal hernias classifies type III and IV hiatal hernias as most severe [49]. Type III hiatal hernias occur when both the fundus and the gastroesophageal junction herniate through the hiatus [49]. Type IV hernias are characterized by an organ other than the stomach protruding through the thoracic cavity [49]. After a mean follow-up time of 14.2 months, there were no recurrences [53]. Complications, such as contraction of repaired defect site leading to recurrence of symptoms, were low with only 8% of patients needing intervention to induce esophageal dilation [53]. Good-to-excellent relief of symptoms, specifically heartburn (20 of 21 patients; 95%) and dysphagia (18 of 19 patients; 94.7%), was achieved in all but one patient, indicating improved quality of life [53].

### 2.3. Inguinal Hernia Repair

Inguinal hernias are defects at the inner groin through which fat or part of the small intestine partially protrude through the lower abdominal wall [54]. As with the aforementioned hernia types, surgical repair is often necessary and the addition of mesh support reduces recurrence rate. These hernias are notable for a high incidence of chronic pain after surgical repair, known as chronic postoperative inguinal pain (CPIP) [55]. While the etiology of CPIP is unknown, it is hypothesized that repair with synthetic mesh causes high inflammation, which irritates surrounding nerves leading to increased pain sensation [56]. Use of a reinforced tissue matrix, such as OviTex, may therefore help to reduce CPIP after inguinal hernia repair. In a single surgeon study, Ferzoco investigated whether use of OviTex in inguinal hernia repair was effective in repairing the defect, reducing recurrence, and preventing chronic postoperative pain (Table 1) [56]. Thirty-one (31) patients, treated on an outpatient basis, underwent inguinal hernia repair with OviTex. There were no reported surgical site infections (SSI) during the initial 30 days postoperatively. At an average 12.6 month follow-up there were no reported recurrences [56]. Additionally, no patients suffered postoperative complications, including onset of CPIP, and there were no requests for postoperative narcotic refills [56]. OviTex seems especially effective in repairing inguinal hernias, as it is not associated with recurrence and its low inflammatory response may prevent occurrence of chronic postoperative pain [56].

## 3. Discussion

Hernia surgery has evolved over the last decade with new techniques that allow for primary closure of fascia under minimal tension. These procedures have shifted from using the mesh as a plug or patch to using the mesh to offload or splint the primary fascial repairs. This provides support of the defect area offloading abdominal pressures and allowing the repair to heal without re-herniation. While the technique and many mesh materials have been developed, the incorporation of polymeric materials still results in inflammation, foreign body response, and complications or recurrences [30,57,58]. Biologic materials have been shown to have lower inflammatory responses, though over time they may stretch and bulge or herniate [30].

The ideal mesh would have the ability to remodel or regenerate into tissue similar to native fascia, without generating a chronic foreign body response resulting in stiffening, contraction, and fibrotic scar tissue. This mesh would also need to possess the proper strength and compliance to maintain the integrity of the repair. A polymeric scaffold that possesses signaling molecules to orchestrate host immune cells and fibroblasts to regenerate human fascia would be the ideal solution. However, it needs to be capable of offloading significant tension while undergoing the remodeling process. Knitted and other polymeric scaffolds either lack the compliance properties necessary for proper offloading in the short-term healing phase [38] or the signaling molecules to guide fascial tissue regeneration in the long term [24]. The incorporation of a polymer within the ECM of reinforced tissue matrices has offered a promising alternative.

The polymer, interwoven through layers of the biologic component of RTMs, provides a reinforcement structure for the repair. This reinforcement is engaged at pressures defined by Junge et al. to mimic the compliance of the abdominal wall, between 11 and 32% [37,38]. The compliance of RTMs has been characterized between 11 and 15% to match the compliance of the native tissue, which in turn transfers abdominal pressures to the mesh rather than the repair site [38]. This reinforcement and offloading of the repair is especially important in patients who have poor collagen formation. The increase in collagen III in the ratio of collagen I and III has been shown to reduce the mechanical strength of connective tissues and may contribute to hernia recurrence [59,60]. If a patient suffering from this is treated with a biologic that is expected to remodel into host collagen over time, the repair may be replaced with poor host collagen, thus decreasing the strength over time from surgery. The polymer structure in RTMs provides a reinforcement network to avoid this and offload the wound, which has yielded low recurrence rates at both the short term and, per early reports, the long term, indicating a promising repair [46,53,56]. This is further evidenced by the results of the Parker ventral hernia repair series evaluating complex patients with lower recurrence rates than other similar studies [23,61,62].

**Figure 3:**
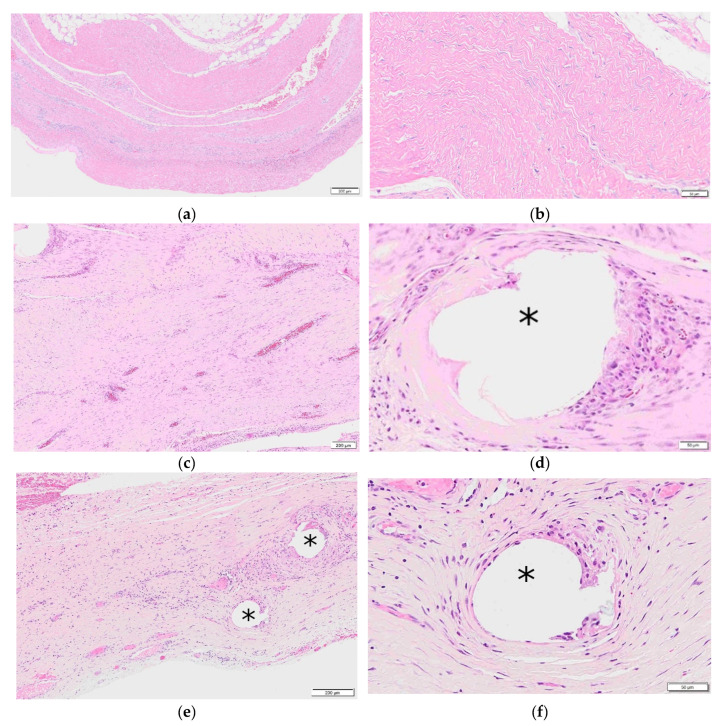
RTM clinical biopsies. * Denotes polypropylene fiber; (**a**) 6 month RTM clinical biopsy, scale bar is 200 µm; (**b**) 6 month RTM clinical biopsy, scale bar is 50 µm; (**c**) 7 month RTM clinical biopsy, scale bar is 200 µm; (**d**) 7 month clinical biopsy, scale bar is 50 µm; (**e**) 23 month RTM clinical biopsy, scale bar is 200 µm; (**f**) 23 month RTM clinical biopsy, scale bar is 50 µm.

The ECM acts as a signaling device to promote host cellular infiltration within the scaffold and promote remodeling of the matrix to attenuate the foreign body response to the polymer and generate functional collagen [24]. The result has shown a low inflammatory response similar to a biologic, lower than that observed in permanent and resorbable synthetic devices [24]. This recruitment of host cells and remodeling is attributed to the porous structure of the ECM and its retention of 153 unique matrisome proteins in the ECM of RTMs [24,40]. The remodeling properties of RTMs have also been observed in patient clinical biopsies, as depicted in Figure 3a–f. The RTM at 6 months, 7 months, and 23 months exhibited low inflammation and end-stage remodeling with the repair exhibiting mature, lamellar, and aponeurosis-like connective tissue. Furthermore, the polypropylene fibers of the 23 month biopsy were immediately surrounded by host cells and tissue with minimal inflammation. It is believed that the embedment of the polymer in a neovascularized biologic tissue allows for direct contact of the polymer with host immune cells, offering the mesh protection from contamination and infection. The ECM combined with the polymeric reinforcement offers a hybrid device providing short- and long-term strength to resist recurrence and a minimal foreign body footprint to limit complications and infections. The results continue to be confirmed in an ongoing 24 month clinical study.

## 4. Conclusions

This review provides an early assessment of the clinical utility of RTMs in an assortment of hernia repairs, where synthetic mesh has historically been used, including high-risk complex ventral hernias and minimally invasive procedures. Use of RTMs results in a decrease in chronic postoperative inguinal pain, improvement of preoperative hiatal hernia symptoms, and a lower incidence of recurrence compared to use of synthetic mesh in a more challenging ventral hernia repair population. These early, yet positive, results are attributed to the embeddment of polymer within a decellularized extracellular matrix, which allows for the construct to be remodeled and form new tissue around the filaments as part of a pro-healing response. The results of the use of OviTex in treatment of hernias continues to be monitored in clinical trials and preclinical studies.

## Figures and Tables

**Table 1:** Follow-up data and results of various surgical hernia repair studies where RTMs were used for repair.

Study	Parker et al., 2020	Sawyer 2018	Ferzoco 2018
**Number of Patients**	50	25	31
**Hernia type**	Ventral	Hiatal	Inguinal
**Hernia severity**	68% modified VHWG grade 3, 70% CDC wound class > I	56% CDC wound class > I	N/A
**Months to follow-up**	12	14.2	12.6
**Surgical site occurrence (SSO)**	36%	N/A	0% at 30 days
**Recurrence rate**	6%	0%	0%
**Postoperative pain**	N/A	6/7 symptoms resolved between 85.7 and 100%	0%

## Data Availability

No new data were created or analyzed in this study. Data sharing is not applicable to this article.
